# Influences of Synchronized Metronome Training on Soccer Players’ Timing Ability, Performance Accuracy, and Lower-Limb Kinematics

**DOI:** 10.3389/fpsyg.2018.02469

**Published:** 2018-12-07

**Authors:** Louise Rönnqvist, Rachel McDonald, Marius Sommer

**Affiliations:** Department of Psychology, Umeå University, Umeå, Sweden

**Keywords:** timing-training, sensorimotor, kinematics, soccer, cognitive-load

## Abstract

Planning and performance of all complex movement requires timing, integration, and coordination between sensory-perception and motor production to be successful. Despite this, there is limited research into “if” and “how” timing training may influence movement performance in athletes. The aim of the present study was to investigate the effect of synchronized metronome training (SMT) on sensorimotor timing ability, and in view of that, if improved timing may be transferred to lower-limb movement planning, precision performance, and kinematics. The sample consisted of 24 female elite- and semi-elite soccer players, randomly assigned to receive SMT and a control group. The SMT group received 12 sessions of Interactive Metronome^®^ (IM) training over 4 weeks. At pre- and post-test, timing-precision was assessed through hand and feet movement synchronization with rhythmic sound; and leg-movements performance accuracy, duration, and kinematics were recorded during embodied high cognitive-load stepping task (6 trials×20 s) by use of a optoelectronic motion capture system. Pre- to post-test comparisons showed significant timing improvements as an effect of the IM training. Significant pre- to post-test improvements on the stepping task performance were seen in an increasing number of accurate foot taps during the stepping task sequence and by shorter duration for the SMT-group only. No evident pre- to post-test effects of SMT on the kinematic parameters investigated were found. These findings signify that the guided attention and working-memory functioning may be positively affected by SMT training; thereby, resulting in better motor planning, performance, and movement precision. Still, independent of group and test-occasion, significant correlations were found between the participants’ outcome performance differences and the kinematic parameters. It was found that a decreasing 3D movement distance and less segmented movements correlating negatively, and increasing velocity (speed) positively, with accuracy and performance duration, respectively. These findings are likely associated with inter-individual variations in the nature of higher-order cognitive processing capacity due to the highly cognitive demanding stepping task.

## Introduction

Synchronized sensorimotor timing skills are important for all daily actions, not least vital for athletic performance, where the smallest mistake in movement can be costly, and only a few milliseconds can make the difference between triumph and failure. Undeniably, timing is especially essential in fast-changing and complex situations and stimuli; as in soccer games. The players have just a few tenths of a second to anticipate where the ball will land, how to position their bodies, decide their next move and speed, be aware of the team-member’s and opponent’s positions and (possible) moves, and decide at what angle and force to direct their next return. These actions are all about timing, planning, sensory-motor synchronizations, coordination and control. The question then is if it possible to train the timing ability? There are convincing numbers of scientific studies that have confirmed positive effects of synchronized metronome training (SMT) for improvements of timing and performance, although surprisingly few includes athletes. In this study we will investigate the possible transfer effects of timing training on soccer player’s lower-limb performances.

The Interactive Metronome^®^ (IM) training program is a specific method of SMT. IM is a unique training tool that challenges sensory and motor systems simultaneously as the individual synchronizes movements with a steady auditory rhythm. Millisecond feedback for mental/interval timing is provided following each movement to help the individual refine and better their sensory-motor timing ability. Several lines of research have shown evidence that after IM training one can see improvements in numerous sensorimotor and cognitive performances (Interactive Metronome^®^ [Equipment], 2012; McGrew, 2013). It is suggested that IM training promotes synchronization within the neural network for more coordinated movement through a series of progressive exercises. Thus, in line with research finding that listening to rhythmic sound activates the centers of the brain involved in coordinated motor planning and sequencing (Bengtsson et al., 2009), and influences internal timing for coordinated movement (Jantzen et al., 2007). Additionally, that action both enriches and complicates the interpretation of sensory inputs and that sensorimotor event may also be transmitted from motor to sensory areas (Merchant and Yarrow, 2016).

The aim of the SMT is to reduce one’s mean asynchrony, or in other words, improve one’s timing and rhythmic ability. When SMT achieves reduction in asynchrony, it results in improved sensorimotor synchronization (SMS). SMS is most often thought about in regards to music and dance, where one’s ability to coordinate perception and action to a beat are necessary (Repp, 2005; Repp and Su, 2013). However, with the increasing popularity of SMT programs such as IM training alongside the growth of neuroimaging studies, there is a growing area of research that suggests that SMS induces transfer effects that are potentially much wider than just for musicians and dancers. For instance, neuroimaging studies (e.g., fMRI) provides support for the idea that SMT can improve spatial-temporal networks within the brain (Chen et al., 2008; Bengtsson et al., 2009), and thereby, inducing changes in motion performance. Intriguing relationships have also been observed between SMS (“beat synchronization”), variability and neural processing of speech in preschoolers (Woodruff Carr et al., 2014). Positron emission tomography (PET) brain investigations have shown that functional cortico-cerebellar circuits sub-serve differential aspects of rhythmic synchronization regarding rhythmic motor control and rhythmic pattern tracking (Thaut et al., 2009). Additionally, relationships between multimodal timing based integrations, brain intrinsic oscillatory gamma activity and reading skills in adolescents have been shown (Woodruff Carr et al., 2017); these findings suggest that beat synchronization and auditory processing may rely on overlapping neural resources that facilitate temporal precision.

The functionality of the brain’s spatiotemporal network, that is to say the brains internal timing mechanisms is inextricably linked with not only motor performance, but even to executive functions (e.g., Stemmer, 1996; Rossini and Pauri, 2000; Diamond, 2003; Mauk and Buonomano, 2004; Nelson et al., 2013). This relationship between timing, motor performance and executive functions, can most obviously be seen in children diagnosed with developmental coordination disorder (DCD). Children with DCD are described as having difficulty with motor planning and control, timing, maintaining attention, and working memory (American Psychiatric Association, 2013). Furthermore, studies of individuals with cerebellum damage have shown that these individuals often suffer problems in executive functions such as learning and memory (Bellebaum and Daum, 2007; Koziol et al., 2012). These studies provide a sound argument, from an evolutionary perspective, for understanding why these two brain areas would be linked. These neural relationships contribute to the explanation of the positive results that SMT has had on children with attention deficit hyperactivity disorder (ADHD) and reading disorders (Shaffer et al., 2001; Taub et al., 2007), as both disorders involve deficits in executive functioning, and in which traditional interventions target executive functioning. Rosenblum and Regev (2013) have described that timing deficits in children with DCD are found to be manifested by their timing performance of the IM tasks, as well as in the temporal measures of their handwriting performance. It has been suggested that SMT has an effect on choice discrimination, which is also a feature of executive functioning (Diamond, 2003).

Taking into consideration the theoretical knowledge and understanding of neural pathways between timing, rhythmicity, and motor planning and control in the brain, it has been proposed that training participants’ timing and rhythmicity can lead to improved motor planning and performance by strengthening these neural pathways (Libkuman et al., 2002; Sommer and Rönnqvist, 2009; Beckelhimer et al., 2011; Chen et al., 2012; Johansson et al., 2012, 2014). A brief review of scientific articles where SMT was used as an intervention has shown support for this. For instance, Shaffer et al. (2001) found that SMT training led to improvements in attention regulation and motor control in both children with special needs and children with ADHD. Beckelhimer et al. (2011) showed improved upper-limb functionality in two participants who had suffered stroke, and Johansson et al. (2012, 2014) saw improved upper-limb movement in two different case studies of children with a different severity of cerebral palsy. An extensive number of clinical studies have also shown that fixed-tempo by the use of Rhythmic Auditory Stimulation (RAS) improves different aspects of gait timing (e.g., Thaut et al., 1996; Hove et al., 2012), and have described the effectiveness of RAS and its promising therapeutic role of improving different aspects of gait performance in patients affected by Parkinson’s disease (e.g., Thaut and Abiru, 2010; Murgia et al., 2015, 2018; Pau et al., 2016; Dalla Bella et al., 2017; Bailey et al., 2018). It’s suggested that RAS would facilitate the activity of the internal clock, thus dys-functioning in patients with Parkinson’s disease (PD), and thereby help in regulating the fluidity of muscular activation, improving coordination, and facilitating the execution of automatic movements, such as walking in PD (Murgia et al., 2018).

In the field of sport science, although existing scientific research is limited, many professional and amateur sport teams have used the IM training method, and reported that their athletes have improved on skills assessments as well as on educational assessments (Interactive Metronome^®^ performance training, 2001; Notre Dame and the Interactive Metronome, 2011; Massad, n.d.). For example, Massad has described how the IM training program has been used and praised by top PGA tour golfers, American football team *Notre Dame*, and NBA team *Miami Dolphins*. In line with these findings, both general rhythm training and tennis specific rhythm training have been described to have an effect on both rhythmic competences as well as on tennis performance in tennis players (Sogut et al., 2012). Although there are many reports from different sports teams and coaches that the IM training program is effective, there are still a limited number of scientific research publications on the relationship between SMT and athletic performance. From the available empirical research, Libkuman et al. (2002) and Sommer and Rönnqvist (2009) have shown that SMT by means of IM training improved golf shot accuracy and reduced the outcome variability. Additionally, Sommer et al. (2014) found that the effect of SMT may not only improve golf shot accuracy, it may also influence the underlying coordinative structures and the temporal synchronicity of the upper-limb movements during the golf-swing performance. Additionally, in a recent study by Kim et al. (2018), the IM training program was implemented to investigate the associations between brain connectivity (during resting state functional MRI) and golf putting performance. The study outcomes showed that IM training reduced the variability of timing between back swing and ball impact in a two-meter putt. In addition, IM training was found to increase brain connectivity from the cerebellum to the frontal cortex. These findings suggest that optimization of motor timing can promote improved motor learning and putting performance in professional golfers (Kim et al., 2018). A recent study from the authors own lab has investigated the effect of IM training in female soccer players performance on cross-pass kick accuracy, and the associated brain response by fMRI while the players were watching videos with soccer-specific actions (Sommer et al., 2018). The results from this study provided both behavioral and neurophysiological evidence that timing training positively influences soccer skill (cross-pass accuracy) and thereby strengthens the action-perception coupling by means of enhanced SMS abilities. Furthermore, it was found that SMT affects the underlying brain responses by means of evident reduced activation within the cerebellar lobule VI activity (Sommer et al., 2018); the brain areas originate to be important for temporal planning, movement coordination, action recognition, and higher cognition functioning (see Keren-Happuch et al., 2014, for a meta-analysis).

At large, the current scientific literature provides evident support for positive outcomes after SMT in a variety of populations, performances, and functions. The present findings of how IM training seemingly effect brain activity within regions facilitating the action perception coupling, as well as induced positive effects on sport performance could be vital for enhancing timing training techniques for athletes. Still, there is a need for further scientific investigation in order to better understand the underlying mechanisms that lead to better motor performance and cognitive functioning, after SMT in athletes. In the present study our aim was to determine whether IM training would add value to traditional soccer training, and if so, if it would induce positive effects on the soccer players planning ability, performance, and in the organization of kinematic properties of leg movements.

Even if the existing literature and theories supports the mediating effect of timing and rhythmicity in regards to one’s attention, cognitive functioning, and motor control, it is uncertain how these mechanisms work together. Accordingly, the present study aimed to contribute to the literature through using an experimental design to investigate the possible effect of the expected timing improvement on lower-limb performance and kinematics, after 4 weeks of IM training. The relationships between timing, motor performance, and executive functions, are central to the design of the present study. Therefore, the stepping task applied in the present study was specifically designed, not only to be relevant to soccer players, but also to require the use of on-line multi-processing such as, inhibition, updating, and shifting. Thereby, creating a state of high cognitive load (simulating the executive chain of decision makings), similar to a real soccer situation. Inhibition, updating, and shifting are three of the most studied constructs within cognitive psychology, and according to Miyake and Friedman (2012) are required to complete complex tasks. Cognitive load and executive functions are related because they rely on working memory to process information (Johannsen et al., 2013).

Based on previous research findings, we expected that the soccer players who undertook the SMT program would improve their timing ability from pre- to post-test, and as a result, perform more accurate and faster lower-limb movements on the specifically designed stepping task. Additionally, we assumed that SMT may lead to changes in the kinematic properties, possibly by means of a less spatio-temporal segmented (smoother) and more time effective movement trajectories (increasing velocity and shorter movement distance), as to be seen in the 3D kinematics recorded of the soccer players whilst preformed the dual motor-cognitive demanding stepping task.

## Materials and Methods

### Participants

Twenty-four female sub-elite and elite outfield soccer players, who played for teams based in Umeå, Sweden, participated in the present study. At the time of pre-testing the participants’ age ranged from 16.2 to 25.8 years (*M* = 19.5, *SD* = 2.7) and they had between 5.5 and 19 years (*M* = 12.7, *SD* = 2.6) of experience playing soccer. All participants were randomly assigned to either the control group or the intervention group. There were no significant between-group differences for age or experience. All participants partook in normal pre-season training during the study. Additionally, the SMT group received three sessions of 45–50 min IM training weekly during a 4 weeks period after the pre-test. For group demographics see Table 1.

**Table 1:** Participant’s mean age, weight, and height, years of experience (±SD), and the number of elite/sub-elite players in both groups.

	*N*	Age (years)	Weight (kg)	Height (cm)	Experience (years)	Elite-/Sub-elite players (*n*)
SMT	12	18.6 (2.4)	62.5 (5.1)	168.9 (5.0)	12.0 (2.2)	8/4
CTRL	12	20.3 (3.01)	60.7 (6.5)	169.7 (6.05)	13.3 (2.87)	6/6


*N, number of participants.*

### Apparatus

#### Tests of Timing and Rhythmicity

The IM system was used to assess both the control and SMT groups’ timing and rhythm skills at the pre- and post-test sessions. The IM test is run in connection with a Windows based computer program that plays a metronome beat into headphones worn by the participant. The IM assessment used at both pre- and post-test sessions was delivered according to standardized protocol included in the instrument manual (Interactive Metronome^®^ [Equipment], 2012). For the present study, the participants wore headphones to listen to the metronome, contact-sensing gloves on their hands, and they used their feet to tap contact-sensing floor mats. These contact-sensing triggers relay information back to the Windows program about the difference between the time at which a participant makes a tap and the metronome beat. For the purposes of the present study there were two soccer related tasks included in addition to the 14 standard tasks (16 tasks in total). Before starting the tests participants viewed a video demonstration of the 16 tasks. For the tests the metronome was set to 54 bpm, with an inter-onset interval of 1100 ms between each task. The participants received no guide sounds or feedback on their performance. The tests took approximately 20 min to complete. The 16 tasks involve completing both uni- and bilateral hand and foot movements to the metronome beat, e.g., bringing both hands together to clap in time with the metronome. The additional two tasks were a stepping task using both feet to step onto floor sensors in synchrony with the metronome and also to perform a kick with their dominant foot at a sensor placed 50 cm above ground. For the present study IM measures from the pre- and post-test sessions were analyzed. These two measures were the *mean rhythmic ability* and the *mean timing ability* of the participants. The *mean rhythmic ability* measures the inter-response interval, which is measured as how close the participant’s response is to their previous response, and the *mean timing ability* measures the millisecond discrepancy between the metronome beat and the participant’s responses, which describes the participant’s SMS or motor timing skill.

#### The Stepping Task

**FIGURE 1:**
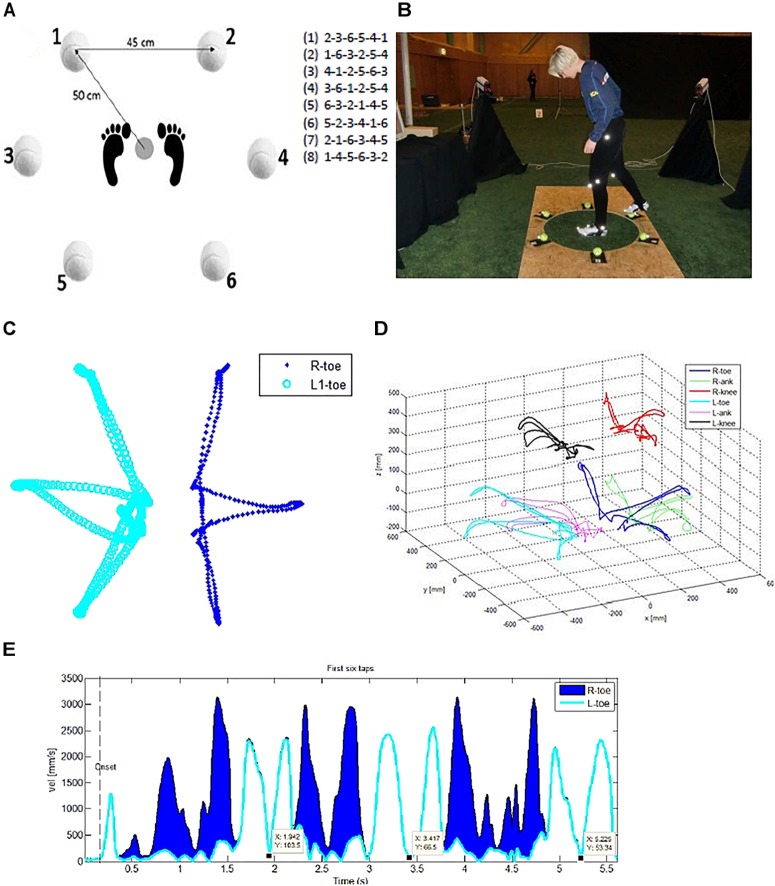
Experimental of protocol illustrating **(A)** the stepping task set-up and example of the eight randomized digit series (e.g., task inducing cognitive-load), contra-balanced (by side), **(B)** photo illustrating the task condition and the marker set-up on the participant, **(C)** example of 2D (x, y) movement trajectories of the right and left foot during the first six step performance (duration 5.7 s) when matching the stepping with the cognitive loaded trial sequence (2/R – 3/L – 6/R – 5/L – 4/R – 1/L) with the actual time position of the three accurate left foot-taps added to the velocity trajectory, and **(D)** the 3D (x-, y-, z-plane) movement trajectories of the right/R and left/L toe, ankle, and knee, respectively, and **(E)** the corresponding velocity trajectories of the right and left toe movements, respectively. The black dots on the velocity trajectory (L-toe) donate the exact moments [time (x) and velocity (y)] of the respectively first three left-foot target hits. Written permission for publication of the photo **(B)** has been reserved from the participant.

Figures 1A,B depicts the experimental lay-out of the stepping task. The instructions that each participant was asked to follow are summarized here. Firstly, the participants were asked stand in the center of a circle of fixated tennis balls, and then to tap the markers with their feet in accordance with a specific digit-series, as many times as possible, while maintaining accuracy, within a 20 s timeframe. The digit-series contained the digits 1–6 (e.g., 1-6-5-2-3-4) and were projected on the screen (placed 2.5 m from the participant at eye level) in front of them throughout the trial, so that they could refer back to the sequence at any time if they forgot the sequence. In other words, the participant had a 20 s period to repeat the sequence as fast and accurate as possible. The participants were instructed to use their left foot to touch markers 1, 3, and 5, and their right foot for markers 2, 4, and 6. Each marker’s number was clearly marked and visible for the participant. Before start, it was emphasized that they needed to not only move as fast as possible, but also to ensure that they minimized mistakes by being precise and accurate. Eight different digit-series were presented, in a randomized, counterbalanced order, and presented after one another, with a small pause (approximately 10 s) between each trial. The presentation onset of the digit-series during the experimental conditions was time synchronized with the onset of the movement registrations. Before the actual start of the experimental session, two 20 s warm-up trials were completed by each participant at both pre- and post-tests. Motion data from the two warm-ups were not measured.

#### Recording and Data Collection

For each trial, the participant’s performance was recorded both in 2D and 3D (illustrated in Figures 1C,D). The 3D movement data was captured with an Optoelectronic motion capture system (ProReflex^®^ [Equipment], 2008) with four cameras, placed around the player, at a distance of 3.5 m from the center of the tennis ball set-up. The 2D recording was captured with a standard video camera placed in front of the participant recoding at 30 fps. The four camera motion capture system by Qualisys Inc. tracks the real-time position (X, Y, and Z, coordinates) and orientation data in 3D space of small, reflective markers attached to the participants (illustrated in Figure 1B). Each of the six tennis balls had reflective markers attached; these markers provided exact information about the time, precision and accuracy of the participant’s foot contact with the ball during the stepping task. The sampling frequency was preset to 120 Hz for all trials, and the quoted accuracy for this system is better than 1 mm for position and 1° for orientation. The cameras were linearized and the system calibrated before the measurement sessions began.

A total of 16 spherical Velcro ProReflex markers were attached to each participant before the stepping task, each marker had a diameter of 7–10 mm. These were placed on each of the hips, knees, ankles, and feet, of the players. Anatomical position is described in Table 2, and illustrated in Figure 1B. Participant’s completed the task with soccer boots on, and so markers for the feet were attached at three positions on each of the soccer boots, other markers were attached to a pair of MoCap pants which have Velcro compatible fabric. In addition, markers were placed on the far side of each tennis ball to aid the 3D analysis (see Figure 1). Six of these 16 markers were selected from each participant to be used in the kinematic analyses. Due to technical difficulties at data collection, it was not always possible to take the same six markers from each participant. However, as markers from the knees (2) and feet (3) were positioned in clusters, tests on the data extracted from the Qualisys software showed that there were no significant differences in the average displacement or velocity profile between each of these markers within one cluster. Thus, one marker with the best data quality (without missing values) was selected from within these clusters. The right and left hip-markers were not included in the analysis as a consequence of inconsistent measurement quality due to temporarily hidden hip-markers.

**Table 2:** Labels and anatomical positions of ProReflex Markers.

Label used	Anatomical position	Segment
Left/right foot	(1) Lateral of the base of the fifth metatarsal	Forefoot
	(2) Medial side of the distal phalanx of the liallux (toe)	Hallux
	(3) Proximal base of the first metatarsal	Foot
Left/light ankle	(4) Lateral calcaneus	Hind foot
Left/right knee	(5) The most prominent part of tuberositas tibiae	Thigh/shank
Left/right hip^∗^	(6) Lateral epicondyle	Shank


*^∗^Not included in the analyses.*

### Procedures

The testing and intervention took place 2 months before the start of the competitive Swedish soccer season. Although not playing games during the study time, all participants participated in their regular team trainings under the test period. At the pre-test session all 24 participants completed a screening questionnaire. At the pre- and post-tests all participants completed both the IM assessment of rhythm and timing and stepping task.

Both pre- and post-testing of the IM assessment and the stepping task took place under controlled and similar conditions for all participants. The IM assessment and training sessions took place in a specially designed lab-setting for this study; the kinematic assessment of the stepping-task took place on an indoor soccer field (with artificial turf) with controlled temperature and lighting. The participants wore similar clothes and soccer boots, the testing areas were blocked off with black curtains to minimize distractions, and all experimental protocols were the same. There was a 100% retention rate, with all participants completing both the pre- and post-test sessions, and those in the intervention group completed all SMT training sessions.

### Ethical Considerations

All participants signed an informed consent form prior to participation. This study is part of a larger research project investigating foot-ball player’s performance as regards to the effects of different training methods and assessments, which has received ethical approval by the Umeå Regional Ethical Board (2011/394-31), and conducted in accordance with the Declaration of Helsinki.

### The Intervention

Participants in the SMT group received three individual sessions per week of IM training provided over a period of 4 weeks, conducted by a qualified instructor. Each training session lasted 45–50 min, resulting in each participant receiving 9–10 h of IM training between the pre- and post-test assessments. Unlike the IM test, in the training sessions the participants receive feedback on whether their hand/foot contact with contact-sensing triggers was on beat, early, or late. This feedback is provided by guide sounds in the headphones worn by each participant. An on beat contact is defined as being within 15 ms of the metronome, and a high pitched tone is simultaneously played into both ears of the headphones. An early contact, one that is 16 ms or earlier before the metronome results in a low pitch tone in the participant’s left ear only. Meanwhile, a late contact, 16 ms or later than the metronome results in a low pitch tone in the participant’s right ear only. This feedback is almost instantaneous and guides the participant to make their movements in time with the metronome through allowing them to adjust and correct their errors continually throughout the IM training session. Each training session began with three or four basic warm-up tasks, i.e., clapping both hands together, or a one hand tap to the thigh, and then moved on to the 32 intervention tasks which consisted of both lower-limb and upper-limb movements, many of the movements were designed to employ soccer-specific skills. The metronome speed varied during the training sessions from 54–100 bpm. At the end of each session the participant would receive a breakdown of their performance as a form of feedback that would motivate them to improve on their scores at the next session. After the 12 training sessions each participant in the SMT group had typically engaged in approximately 27000 motor repetitions.

### Data Extraction and Analysis

To establish the training effects from the IM intervention, the participant’s timing ability and rhythmic ability (measured in milliseconds) from the pre- and post-test sessions were extracted from the IM computer program.

To illustrate the player’s performance on the stepping task, two variables were extracted from the 2D video recording, and conferring with the synchronized 3D optoelectronic measures of the pre- and post-test. These variables were number of correct foot taps and time taken (duration) to complete first six taps. Each trial was first analyzed one-by-one to identify and count the total number of successful taps made by the participant and to assess how many of these taps matched the specific digit-series they were instructed to follow for that trial. A successful tap was defined by the soccer boot making clear contact with the tennis ball, taps beside or behind the ball, which did not make contact with the ball were not counted as successful. Due to technical issues during the testing session, not all trials for all participants were recorded correctly and therefore there are some missing data. In total, two trials (or 1% of all pre-test data) from the pre-test (both from SMT group) and four trials (or 2% of all post-test data) from the post-test (three from SMT and one from control) are missing.

Due to the variation in total number of taps made by each participant in the 20 s stepping task the kinematic data analyses was extracted from each trial of the participant’s first six taps. The kinematic data from each camera were first transformed into three-dimensional (X, Y, and Z) coordinates by the system software (Qualisys Track Manager). All kinematic data was smoothed prior to analyses using a second-order 10 Hz dual-pass Butterworth filter, and analyzed offline with custom written software in MATLAB (The Mathworks Inc.). The kinematic variables that were extracted for statistical analysis were the cumulative 3D distance (mm), the total number of movement units (MU), and the average velocity (mm/s). Cumulative 3D distance is the total accumulated distance extracted from each marker to gain insight into the energy efficiency of the movement paths (exemplified in Figures 1C,D). Movement units describe the segmentation of the movement trajectory and are based on tangential velocity and acceleration data (exemplified in Figures 1E,F). The algorithm for calculating MUs was taken from previous studies of movement segmentation by von Hofsten (1991) and Rönnqvist and Domellöf (2006), where one MU is defined as a change from an acceleration or deceleration phase with a rate of change in accumulated velocity of at least 20 mm/s and change in acceleration/deceleration of at least 5 mm/s^2^.

The total number of successful kinematic recordings extracted for further analysis was from in total 289 (20 s) stepping trials that represented 76% of all pre- and post-test data. In total 94 trials (24%) of the kinematic recordings were excluded because of poor recording quality. This was due to markers not being measured properly; e.g., >10 frames missing in a row, markers hidden, and/or marker drop-off. Small marker omissions (<10 frames) were pre-set to be gap filled by the system software. All automatic gap fills were visually inspected and adjusted in cases where the fill was deemed incorrect before accepting them. Larger gaps in the recordings (maximum of 20 frames) were manually gap-filled if deemed that it could be done reliably. All larger gap-fills were checked in Z-, Y-, and X-plane, acceleration profile and velocity profile before accepting them.

### Statistical Analysis

Tests of homogeneity of variance, normality, and between-groups *t*-tests supported that the sampled data were approximately normally distributed and that the two groups were equal at pre-test in regards to demographic variables, performance on the stepping test, and in kinematic measures. Further, no significant group difference in timing or rhythmic performance was found at the IM pre-test. Where the assumption of homogeneity was not met, Greenhouse-Geisser corrects were applied. Due to the intervention focus of the study effect sizes (partial eta-squared: $\eta_{p}^{2}$) are provided where applicable, interpretations of these are steered by Cohen’s (1969) guidelines: $\eta_{p}^{2}$ > 0.01 or 1% = small effect, >0.06 or 6%, medium effect, and >0.14 or 14% = large effect.

To analyze the effect of SMT on the soccer players’ timing ability and lower-limbs movement performances in the stepping task, separate mixed analysis of variance test (ANOVAs) with repeated measures were used, with group (SMT – control) as the between-individual factor and “test” (pre-test – post-test) as repeated within-individual factor. Each dependent variable was run in separate tests. Regarding the kinematic variables (cumulative 3D distance, number of MUs, and the average velocity correspondingly) a composite value was calculated from the knee, ankle, and toe markers for the respective right and left leg kinematics. Significant between group and time interactions was followed up by use of *Scheffe’s post hoc* comparison. *Pearson* product-moment correlation coefficient was used to explore the relationship between the players’ timing ability (mean response time; difference in milliseconds between the moment the beat sounds and the participant’s motor response, with longer response time indicating lower time management ability) and the movement performance (number of correct foot taps and duration) and the kinematic outcome parameters (MUs, 3D distance, velocity) from the stepping task. A pre-set alpha level of 0.05 was used as the level of significance for all statistical tests. All statistical analysis was made by using the STATISTICA software (StatSoft 11^1^).

## Results

### Timing Ability

**FIGURE 2:**
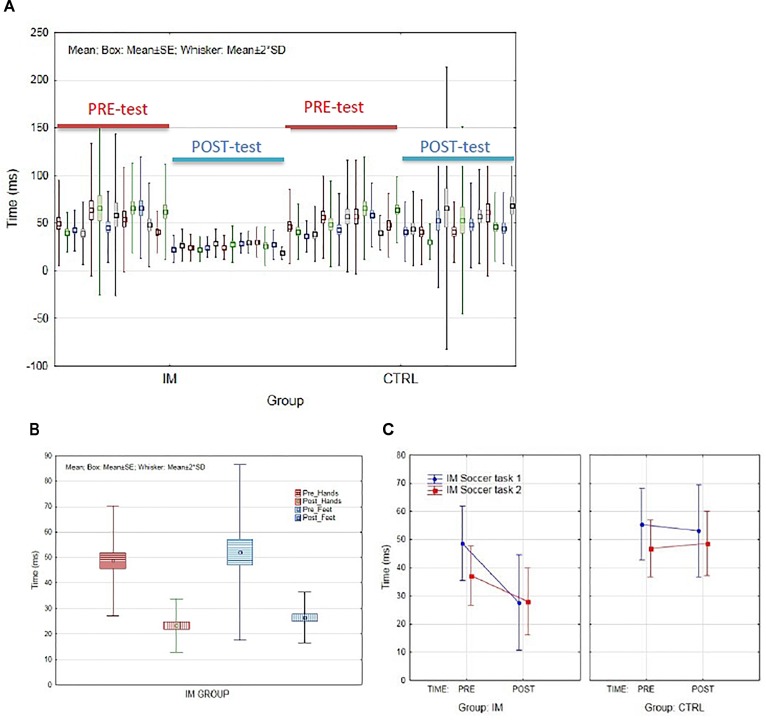
The pre- and post-test mean response time in milliseconds from **(A)** all the fourteen standard IM timing tasks for the SMT group and the control group, respectively, and **(B)** the mean response time from the two IM categories including arms-hands (tasks 1–3, and 14), and legs-feet (tasks 4–9, 12, and 13) as an effect of tests for the SMT group only, and **(C)** the mean response time from the two soccer related tasks (1, stepping task including both feet, and 2, kicking task with the dominant foot) as a function of tests and groups.

**FIGURE 3:**
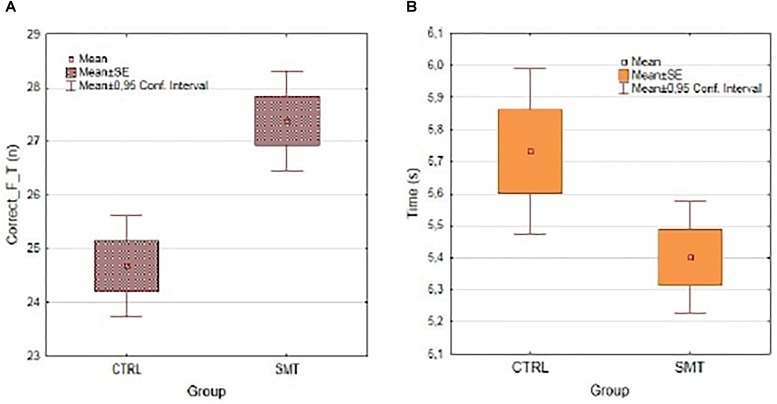
Presenting **(A)** the mean number of correct foot taps during the 20 s stepping task as a function of groups at the post-test comparison, and **(B)** the mean duration to complete the first six steps of the stepping task as a function of group at the post-test comparison.

To analyze the intervention effect of SMT on timing and rhythmic ability (mean response time including all the fourteen standard IM-tasks in milliseconds) were statistically analyzed. The pre- and post-test outcomes from all 14 tasks for respective group are presented in Figure 2A. The ANOVA revealed a significant main effect for group; *F*(1,22) = 5.24, *p* < 0.05, $\eta_{p}^{2}$ = 0.19, a significant effect of test; *F*(1,22) = 21.83, *p* < 0.001, $\eta_{p}^{2}$ = 0.50, and a significant group by test interaction; *F*(1,22) = 21.40, *p* < 0.001, $\eta_{p}^{2}$ = 0.49. Partial eta-squared showed that 49.3% of the variance in the improvement of the participants’ timing ability can be related to the IM intervention. A second ANOVA was performed to test the impact of the IM intervention on the participants’ mean rhythmic ability. In line with the outcome from the timing ability, the ANOVA revealed a significant main effect for group; *F*(1,22) = 8.20, *p* < 0.01, $\eta_{p}^{2}$ = 0.27, a significant effect of time; *F*(1,22) = 17.60, *p* < 0.001, $\eta_{p}^{2}$ = 0.44, and a significant interaction between group and test; *F*(1,22) = 14.91, *p* < 0.01, $\eta_{p}^{2}$ = 0.40. Partial eta-squared showed that 40.4% of the variance in the improvement of the participants’ rhythmic ability can be related to the IM intervention. The *Scheffe’s post hoc* comparisons revealed a significant pre- to post-test improvement for both the timing and rhythmic ability, respectively, for the SMT group (*p* < 0.00). No significant pre- to post-test improvements were found for the control group (timing: *p* = 0.99; rhythmic: *p* = 1.0). As illustrated in Figure 3A the variability was evidently decreasing from pre- to post-test for the SMT group. Figure 2B highlights the mean response time for the two IM categories including hands (tasks 1, 2, 3, and 14) and feet’s (tasks 4–9, 12, and 13), at the respective pre- and post-test for the SMT group only. To analyze the effect of SMT on the timing ability (mean IM response time) for the two specially designed soccer related tasks for this study (1, a stepping task using both feet and 2, a kicking task with the dominant foot) a separate ANOVA was used. The ANOVA revealed no significant main effect for group; *F*(2,22) = 3.34, *p* = 0.054, $\eta_{p}^{2}$ = 0.15, no significant effect of test; *F*(2,22) = 3.39, *p* = 0.051, $\eta_{p}^{2}$ = 0.15, yet a significant interaction between group and test; *F*(2,22) = 3.86, *p* < 0.05, $\eta_{p}^{2}$ = 0.16. As presented in Figure 2C, the SMT group showed significant improvement by shorter response time at the post-test during the two soccer related IM task, improvements were not found for the control group.

### Performance Accuracy and Duration

The ANOVA completed for investigating the effect of improved timing ability on the number of correct taps during the 20-s stepping task revealed no significant main effect of group *F*(1,186) = 3.21, *p* = 0.07, $\eta_{p}^{2}$ = 0.02, a significant effect of test *F*(1,186) = 25.4, *p* < 0.001, $\eta_{p}^{2}$ = 0.12, and a significant group by test interaction *F*(1,186) = 5.20, *p* < 0.05, $\eta_{p}^{2}$ = 0.03. The *Scheffe’s post hoc* comparisons confirm that the SMT group significantly improved by means of the number of correct foot taps from pre- to post-test (*p* < 0.001), no such significant improvement was found for the control group (*p* = 0.24), see Figure 3A. Regarding the duration to complete the first six steps sequence during the stepping task, the ANOVA revealed no significant main effect for group *F*(1,135) = 1.82, *p* = 0.18, $\eta_{p}^{2}$ = 0.01, no significant effect of test; *F*(1,135) = 1.96, *p* = 0.16, $\eta_{p}^{2}$ = 0.01, yet a significant interaction between the test and the groups *F*(1,135) = 4.64, *p* < 0.05, $\eta_{p}^{2}$ = 0.03. As illustrated in Figure 3B, the duration of the first six foot taps was significantly shorter (*p* < 0.05) for the SMT-group at the post-test in comparison to the Control-group who did not show any significant pre- to post-duration change (*p* = 0.28).

### Kinematic Outcomes

#### Number of Movement Units (MUs)

The ANOVA completed to analyze the pre- to post effect on the number of MUs (movement segmentations) made during the first six foot taps sequence (of the 20 s stepping trial) as a function of group and test revealed no significant main effect for group; *F*(1,120) = 1.37, *p* = 0.24, $\eta_{p}^{2}$ = 0.01, a significant effect of test *F*(1,120) = 8.03, *p* < 0.01, $\eta_{p}^{2}$ = 0.06, and no significant interaction between test and groups; *F*(1,120) = 1.38, *p* = 0.24, $\eta_{p}^{2}$ = 0.01. This result shows that independently of groups, a significant increasing number of MUs was found at the post-test (*M* = 38.4, *SD* = 6.8), in comparison to the pre-test (*M* = 36.2, *SD* = 9.6).

#### Cumulative 3D Distance

The ANOVA completed to analyze the pre- to post-test effect of the cumulative 3D distance revealed no significant main effect of group; *F*(1,120) = 0.08, *p* = 0.76, $\eta_{p}^{2}$ = 0.001, a significant effect of test *F*(1,120) = 9.71, *p* < 0.01, $\eta_{p}^{2}$ = 0.07, and no significant interaction between test and groups *F*(1,120) = 1.44, *p* = 0.23, $\eta_{p}^{2}$ = 0.01. This result shows that independent of group, a significant shorter 3D movement distance was found at the post-test (*M* = 270.3 cm, *SD* = 30.6 cm) in comparison to the pre-test (*M* = 288.9 cm, *SD* = 33.4 cm).

#### Average Velocity (mm/s)

The ANOVA completed to analyze the pre- to post-test effect of the average velocity revealed no significant main effect of group *F*(1,120) = 2.95, *p* = 0.09, $\eta_{p}^{2}$ = 0.02, a significant effect of test *F*(1,120) = 9.49, *p* < 0.01, $\eta_{p}^{2}$ = 0.07, and a significant interaction between test and group *F*(1,120) = 4.05, *p* < 0.05, $\eta_{p}^{2}$ = 0.03. This result shows that independent of group, a decreasing average velocity was found at the post-test (*M* = 506 mm/s, *SD* = 0.79 mm/s), in comparison to the pre-test (M = 482 mm/s, *SD* = 0.60 mm/s). In addition, the *Scheffe’s post hoc* comparisons confirmed a significant difference (*p* = 0.04) between the SMT and the control group; whereas only the control group showed a significant decreasing velocity profile at the post-test (*M* = 460 mm/s, *SD* = 63 mm/s), in comparison to the pre-test (*M* = 504 mm/s, *SD* = 81 mm/s).

#### Associations Between Outcomes

As would be expected, a strong significant correlation between timing and rhythm performance outcomes was found (pre-test; *r* = 0.895; post-test; *r* = 0.910), this is because these are two related measures of the overall motor timing ability.

A significant correlation was observed between the IM response time and the number of correct foot taps performed (based on individual mean number of correct foot taps over 20 s task interval) for the SMT group (*r* = -0.557, *p* < 0.01), indicating that a decreasing IM response time associates with an increasing number of correct foot taps at the post-test. There were no statistical significant correlations found for either group regarding the relationship between the IM response time and the kinematic outcomes variables from the stepping task.

However, when the correlations between the IM response time, stepping performance, and kinematic outcomes (MUs, 3D distance, and velocity) are based on individual task performances independent of group, some interesting associations appears. As presented in Table 3, a significant negative correlation was found between the IM response time (timing ability) and the number of correct foot taps among the soccer players at both the pre- and post-test (*r* = -0.486, *p* < 0.05; *r* = -0.588, *p* < 0.01). This correlation indicates that the participants with a longer response time (poorer timing ability) are those who preform less correct foot-taps at both the pre- and the post-test. In addition, at the post-test a significant positive correlation was found between the IM response time and the six steps duration (*r* = 0.700, *p* < 0.01), indicating that a shorter response time (better timing ability) at the post-test links with a shorter step duration. In addition, a significant negative correlation between the IM response time and the average velocity (*r* = -0.458, *p* < 0.05) was found, indicating that better timing ability is also related with faster movements.

**Table 3:** Correlations between the IM response time (means based of 14 IM standard tasks) and stepping-task parameters (means based on number of correct foot taps/20 s, and duration of the first six foot taps) and kinematic measurements (MUs, 3D distance, and velocity), independent of group.

	IM response time	
	
Parameters	Pre-test	Post-test
Correct F_T (n)	-0.486^∗^	-0.588^∗^
Duration Six F_T (s)	0.373	0.700^∗∗^
MUs (n)	0.116	0.362
Distance (mm)	0.228	0.337
Velocity (mm/s)	-0.244	-0.458^∗^


*^∗^*p* < 0.05, ^∗∗^*p* < 0.01.*

**FIGURE 4:**
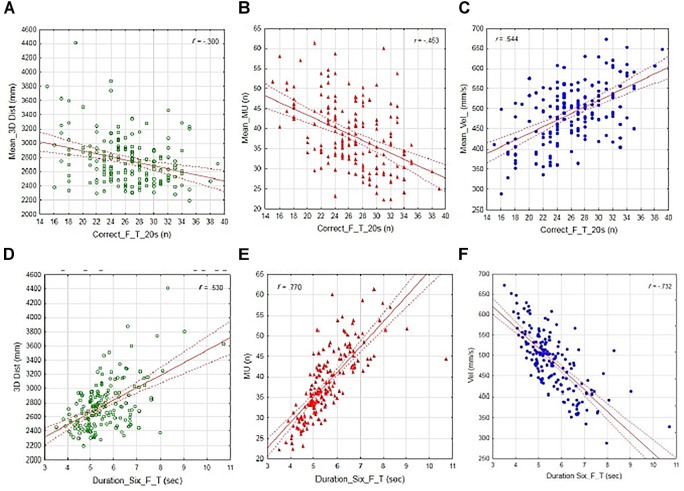
Scatter plots of the relationship between the two respective stepping performance variables: mean number of correct taps during 20 s **(A–C)**; mean duration of the first six taps **(D–F)**, and the kinematic variables: 3D distance, number of MUs, and average velocity. The dotted lines represent 95% confidence intervals.

Independently of groups, correlations between stepping performance (number of correct foot taps and duration) and the kinematic outcome variables based on individual trials at post-test are presented in Figure 4. Significant correlation was found between number of correct foot taps and (a) 3D distance (*r* = 300 *p* < 0.05); (b) number of MUs (*r* = 453, *p* < 0.01); and (c) average velocity (*r* = 544, *p* < 0.01). Significant correlations were also found between the duration of the first six foot taps and (d) the 3D distance (*r* = 530, *p* < 0.01); (e) the number of MUs (*r* = 770, *p* < 0.001); and (f) the average velocity (*r* = -732, *p* < 0.001). These findings of significant correlations indicate that the increasing number of correct foot taps during the stepping task is related to shorter 3D movement distance, less MUs, and a higher average movement velocity. Moreover, that a decreasing performance duration of the first six foot taps is associated with a shorter 3D distance, less MUs and increasing movement velocity. Similar significant correlations and relationships between kinematics and stepping task performance were also found at pre-test when based on all individual trial outcomes (independently of groups).

## Discussion

There is a growing body of studies confirming that timing and rhythmicity training programs are beneficial in a variety of rehabilitation settings, clinical conditions, and in affecting a variety of human behaviors. For example, motor planning, sequencing, and cognitive functions, such as academic achievement and attention. Based on these cross-domain findings, the concept of timing training has also gained popularity in applied fitness settings to enhance sport performance. For instance, a recent study where the use of rhythm was used to improve a cycling (motor) task (Bertollo et al., 2015); both external (i.e., focusing attention on a metronome) and internal (e.g., focusing on pacing) strategies were found to be effective. Yet, there are few scientific studies that have investigated the efficacy of synchronized timing training and its effect on sport performance and athletic achievement. As far as we know, there has not previously been any study investigating what possible effect SMT might have on lower-limb performance and kinematics. The present study was designed to determine potential effect of SMT on sensorimotor timing ability, and also, to investigate whether improved timing ability may provide additive value to traditional soccer training.

In line with our expectations, and with previous findings from studies including golfers and soccer players, as well as various groups of clinical populations and children suffering from various impairments, it was found that IM training produced significant improvements in timing and rhythmicity, manifested in reduced response time. This finding confirmed timing improvements also in the highly experienced and physically well-trained soccer players who took part of the IM intervention in this study. This timing improvement was found after just 9–10 h of IM training over a 4-week period. This was seen by means of evident decreasing response time between pre- and post-test. Accordingly, decreasing intra- as well as inter-individual timing variability was seen as an effect of IM training. In addition to this, the two specifically designed soccer-related IM tasks that were added confirmed timing improvements for the SMT group. Thus, these findings support the suggested explanation that improvements on the underlying brain network related to sensorimotor communication and synchronization, and that IM interventions may improve the resolution and efficiency of an individual’s temporal processing (Taub et al., 2007, 2015; McGrew, 2013).

### Effects of SMT on the Stepping Task Performance

It was expected that both groups would improve on the stepping task since, at pre-test the task was novel to all participants, and there was only 4 weeks between the tests. Still it was assumed that the rate of improvement between the two groups would differ, favoring the SMT group. The analysis confirmed this expectation as significant improvements were found for the SMT group only. The SMT group showed a significant, increasing number of correct foot taps and took on average a shorter time to complete the stepping task at the post-test in comparison to the pre-test. These results add to previous findings of improvement in outcome accuracy as an effect of SMT found on golfers’ performance (Libkuman et al., 2002; Sommer and Rönnqvist, 2009); reduced variability and enhanced precision of soccer cross-passes in soccer players (Sommer et al., 2018); and to findings from similar timing training programs that have reported associations between rhythmic competences and improved tennis performance (Sogut et al., 2012).

Because motor planning was a key part to the cognitively loaded stepping task used in this study, it recruited working memory, attention and other executive functions, which are vital to good motor planning ability. Thus, understanding these results within the context that SMT improves motor planning through improving motor timing and rhythmicity (Stemmer, 1996; Rossini and Pauri, 2000; Diamond, 2003; Mauk and Buonomano, 2004; Koziol et al., 2012); the findings from the present study provide support for the explanation that SMT may strengthen the neural pathways within the spatiotemporal network, and thereby improve motor planning ability. Due to the fact that the stepping task introduced in the present study involves simultaneous coordination of a motor and a cognitive task (to learn and process the digit sequence and meanwhile, plan and produce precise, coordinated stepping movements, and in the right order), the positive SMT effect found by means of stepping accuracy (number of correct taps) and decreased duration, may also be explained as increasing efficiency of working memory; thus, that ultimately results in more efficient complex information processing. This explanation is in line with the suggestion that IM-based training may increase the efficiency of attentional and inhibitory control, and thus result in an increase in automatization and efficient performance of working memory (Taub et al., 2015). Thus, suggesting domain-general influences of SMT on the underlying brain-based sensorimotor control and planning strategies may explain the improved lower-limb movement performance found for the cognitive demanding stepping task.

In the present study, we did not confirm previous findings of changes in the kinematic properties and dynamics (found in the golf swing) as an effect of SMT (Sommer et al., 2014). When comparing the pre- to post-test data and possible group differences, no apparent effect of SMT was found from the kinematic data investigated. This study focused on movement segmentation (MUs), 3D distance, and the average velocity, and thus the kinematic variables extracted during the stepping task describe possible changes in lower limbs movement dynamics and efficiency after SMT. One possible interpretation of the lack of kinematic changes in this study is that the working memory resources involved in the processing of the stepping task may interfere with the motor performance during the foot tapping serials. Thus, that the use of the high cognitive loaded task may create substantial higher-order processing competition between the motor and the cognitive domains. This interpretation is in line with the dual-task interference phenomena described by Johannsen et al. (2013), who suggested that the effect of dual-task interference predominantly concerns the timing accuracy of movement control and not the kinematics (findings based on timing of periodic ankle movements). Another possible explanation might be that even if the representation of a timing skill is reorganized in the brain shortly after an internal model has been acquired, this reorganization may not have been established in the kinematic properties; it may still contribute to increased stability of the representation of the sensorimotor timing improvements. Thus, this explanation is in line with the suggested sequential stages of motor memory consolidation (Shadmehr and Holcomb, 1997).

The findings that both the control and the SMT groups showed changes by means of increasing number of movement segmentations (MUs), shorter movement trajectories, and a decreasing performance speed (average velocity) at the post-test may be interpreted as a test–re-test effect. Yet, it may also be considered alongside Bernstein’s theory on skill acquisition in complex movement performance. Bernstein (1967) suggested that the mastery of complex motor skills is dependent upon increased availability of redundant degrees of freedom (DOF); this increase in DOF available to the individual that has mastered a complex movement allows them to become more efficient, and more flexible, in their motor performance. Therefore, the pre- to post-test changes found by means of increasing number of MUs may be interpret as reflecting the availability of more DOF presented as a test-task-training-effect. Thus in line with Verrel et al.’s, (2013) interpretation of the increased availability of DOF in the context of skilled string instrumentalists who need to make fast and efficient changes of direction: a skill also necessary in the stepping task used in the present study. The decreasing performance speed found at post-test for the control group, although with a small effect size, may be explained as a speed-accuracy trade-off effect, usually described as competing demands of response speed and response accuracy (Belkin and Eliot, 1997).

It was expected that there would be a relationship between participants’ pre- to post-test IM response time and the stepping task performance after completing the SMT intervention. In other words, that those who improved most on the IM test (by means of decreasing response time) would improve most in the stepping task. In line with this prediction a significant correlation was found between the IM response time and the number of correct of steps performance. This finding supported previous findings describing the relationship between improved sensorimotor timing skills and improved sport performance and accuracy, as an effect of SMT. Yet, as seen from the results of the pre- and post-test comparison from the ANOVAs, no significant relationships between the IM generated timing improvements and the kinematic outcomes were found for either group. These findings indicated that the effect of SMT had a stronger impact on the soccer players’ sensorimotor planning ability (as a possible result of enhanced executive functions and attention), than on the spatial-temporal aspects of kinematic properties, *per se*. Thus, these results are contrary to previous findings from kinematic outcome analyses based on upper-limb performance during the golf swing; which suggested that SMT may not only effect golf performance accuracy, but also influence the underlying coordinative structures and temporal synchronicity (Sommer et al., 2014). Essentially, these comparisons of kinematics are from two different motor tasks, with different demands, and based on different movement performance and sports.

Even though the present study did not show any evident pre- to post-test effect of SMT on the kinematic properties, some attention-grabbing findings emerge from correlations made between the kinematics (on individual task levels) and movement performance independently of group. These correlations indicate that the increasing number of correct foot taps during the stepping task is related to better timing ability (shorter response time), a shorter 3D movement distance, less movement segmentations (MUs), and a higher average movement velocity. Moreover, decreasing performance duration of the first six foot taps was associated with a shorter 3D distance, and fewer MUs, as well as with increasing movement velocity. These findings are most likely associated with the inter-individual variations in the nature of higher-order cognitive processing capacity in the participants and emphasized due to the demands of the cognitive-load added to the stepping task. In addition, sensory-motor synchronization, coordination, and sequencing during complex actions may be associated with individual variations in the nature of the neuro-anatomical constraints that determine the control of goal-directed lower-limb movements. Recent studies have suggest that executive functions (EFs; the cognitive control processes that regulate thoughts and actions), are critical for high performance in soccer. For instance, it has been demonstrated that elite young soccer players showed higher scores in general EF tests than non-elite youth players (Huijgen et al., 2015; Vestberg et al., 2017). Similar results have also been reported in hockey players (Lundgren et al., 2016). A positive relationship between successful sport performances in young soccer talents and their cognitive creativity in general have also been described by Kovac (1996). Relationships between elite soccer players’ EF and the number of goals they scored two seasons later have also been observed (Vestberg et al., 2012, 2017), leading the authors to suggest a causal role of the soccer players’ cognitive function and their future soccer success. Hence, these findings of EFs and performances are in agreements with the well-established findings of associations between cognitive control processes and timing ability in general based on chronometric tasks; reaction time, inspection time, time discrimination, and interval production (Ullén et al., 2008; Holm et al., 2017). Additionally, neuroimaging data have also suggest that timing tasks load more on cognitive control as they become sensory rather than motor (Lewis and Miall, 2003; Rammsayer and Troche, 2014).

A shortcoming of the present study is that we did not include information outcomes from standardized neuropsychological assessment regarding the participants’ general executive functions; further studies should employ these assessments. Another limitation was that we were unable to include the kinematic recordings from the hip markers due to measurement-technical problems. Furthermore, application of time series analysis of the leg dynamics may give added information of the soccer players’ stability and variability and of the leg dynamics (joint inter-couplings) during the stepping task, as a possible effect of SMT.

### Future Research

It is recommended that further studies should address the effect of SMT on movement performance and kinematics by comparing different levels of athletes with non-athletes, and during different level of task complexity; with- and without cognitive-load(s). This would provide evidence on the effects of SMT in groups with differing levels of experience/training, and therefore provide information on its efficacy in different populations and sports. In addition, more neuroimaging (fMRI) studies to investigate the effect of SMT on structural-functional brain activation and locations in relation to different aspects of performance are requested for. In addition, the possible long-term effects of SMT are fairly unknown and in need of evaluation. Follow up data would provide information on whether the effects of SMT are long lasting, and optimally, provide further important knowledge about consolidation and task-general transfer effects. To elucidate the relationship between cognitive and sport performance, further experiments are needed that directly test the causal relationship between both internal and external timing ability and cognitive performance and EF in athletes.

## Conclusion

Being the first study to utilize 3D kinematic analysis to assess the effects of SMT in the context of soccer player’s leg movements, the present study provides new and meaningful information about the effects of SMT on sensorimotor efficiency, motor planning, and performance. The results support the suggested explanations that improvements of the underlying brain network relate to sensorimotor communication and synchronizations, and that the IM training may improve the resolution and efficiency of an individual’s temporal processing. The results also provide support for the theoretical and empirical link between efficient cognitive capacity and sensorimotor performance and accuracy. To conclude, even if physical strength and mental ability are of high importance in all sport performance, sensorimotor timing ability may be the key factor. The present study emphasizes the importance of improving the cognitive-motor planning ability and sensorimotor timing skills, especially in team sport such as soccer where the players have to rely on accurately timed actions on milliseconds level. It is clear from the results that synchronized sensorimotor timing abilities should be acknowledged as a critical factor for soccer player’s performance. Additionally, that SMT also may be beneficial for both general motor planning and in other sports performance and situations of complex, high cognitive-motor demanded tasks, and dynamic contexts.

## Author Contributions

LR and MS conceived and designed the work, and acquired the data. LR, MS, and RM contributed in analysis and interpretation of the data, drafted the work, revised the work, contributed in final approval of the version to be published, and agreed to be accountable for all aspects of the work in ensuring that questions related to the accuracy or integrity of any part of the work are appropriately investigated and resolved.

## Conflict of Interest Statement

The authors declare that the research was conducted in the absence of any commercial or financial relationships that could be construed as a potential conflict of interest.
